# Reliability and validity of the fall risk self-assessment scale for community-dwelling older people in China: a pilot study

**DOI:** 10.1186/s12877-022-02962-3

**Published:** 2022-04-01

**Authors:** Zhizhuo Wang, Yuetong Rong, Li Gu, Yanyan Yang, Xinmin Du, Mouwang Zhou

**Affiliations:** 1 Department of Rehabilitation Medicine, Peking University Third Hospital, 49 North Garden Road, Beijing, 100191 China; 2 Jimenli Community Health Service Center, North Third Ring West Road, Haidian District, Beijing, 100088 China

**Keywords:** Fall risk self-assessment scale, Older adults, Community, Reliability, Validity

## Abstract

**Background:**

Falls are a common and serious public health issue among older adults, contributing to the loss of independence, psychological distress, and incapability to engage in meaningful occupations, etc. However, there is a lack of abundant information about the fall risk self-evaluation scale for community-dwelling older people. Therefore, this study aimed to evaluate the preliminary reliability and validity of the fall risk self-assessment scale (FRSAS) among community-dwelling older adults.

**Methods:**

A cross-sectional study was conducted. A total of 230 individuals aged 65 years and over were recruited by a convenience sampling between October and December 2020 from three communities in Haidian district, Beijing. Eligible participants were required to fill in the general condition questionnaire and the fall risk self-assessment scale. The reliability and validity were analyzed by using SPSS 20.0.

**Results:**

Two hundred twenty-two participants completed the assessment as required (the completion rate was 96.52%). The most items of FRSAS were understood by older adults, which was completed in 10 min. Cronbach’s α and intraclass correlation coefficient ICC (2,1) of the scale were 0.757 and 0.967 respectively, suggesting good internal consistency and test-retest reliability. Exploratory factor analysis yielded 14 factors that explained 61.744% of the variance. Five items failed to be categorized into any factors because the factor loading of these items was less than 0.4. A future large-sample study needs to be conducted to explore its construct validity. The total scores and dimensional scores except for C-dimension showed significant differences between participants who had experienced a fall in the previous 6 months and those who had not (*P* < 0.05), indicating good discriminant validity.

**Conclusions:**

The fall risk self-assessment scale including 41 items demonstrated relatively high feasibility as well as satisfactory results in the internal consistency, test-retest reliability, and discriminant validity.

**Trial registration:**

Registration number: ChiCTR2000038856; Date of registration: 7 Oct 2020.

**Supplementary Information:**

The online version contains supplementary material available at 10.1186/s12877-022-02962-3.

## Background

Falls are a common and serious public health issue among older adults [1, 2]. It is reported that the annual rate of falls in Chinese community-dwelling older people ranged from 14.7 to 34% and the annual incidence of falling more than once was 4 to 5% [3]. A fall is universally defined as “an event resulting in a person who comes to rest inadvertently on the ground, floor or other lower-level” [4]. Although falling is not regarded as a determinant of an underlying poor health condition, ramifications of falls may predispose individuals to adverse outcomes ranging from minor injuries (e.g., bruises, abrasions, lacerations, strains and sprains) to serious consequences, including fractures, loss of independence or even death [5–7]. Besides physical injuries, psychological problems resulting from falls should not be underestimated, such as fear of falling [8]. Evidence showed that the cost of fall-related injury among older adults ranged from US$16 to US$3812 per person per fall in China [9]. The possibility of falls will significantly increase with the number of fall risks identified, supported by a prospective study with the percentage which fell within 1 year nearly folded with each additional risk factor in community-dwelling older persons [10]. Therefore, assessing fall risks prospectively can curb the fall incidences effectively and decrease the costs of either hospital agency or family.

Although a few fall risk assessment tools are readily available, most have been tested or used in primary care settings [11]. Gate et al. emphasized that tools developed in one setting (e.g., inpatient) may be less efficacious when used in another setting (e.g., outpatient) [12]. Based on that, other fall risk assessment tools have been developed for predicting fall-related risks in community-dwelling older adults. Through searching the database including PubMed, Medline, Embase and China National Knowledge Infrastructure (CNKI), several fall risk assessment tools used in community settings have been identified [13–25]. As shown in Table 1, these assessment tools can be roughly divided into three categories including the performance-based tests, the fall-related psychological evaluations and the multifactorial fall risk assessment tools. Regarding the performance-based tests, they are nearly performed by the healthcare providers, which requires relative longer administrative time and a certain number of qualified staffs. Although the fall-related psychological evaluations can be completed by the older adults themselves, these evaluations just attend to the psychological aspects of fall-related risks, excluding the physical agents. In addition, the fact that the multifactorial fall risk assessment tools are time-consuming, albeit rather comprehensive, suggests that they are rarely incorporated into the daily routine of community-dwelling older adults to predict the risk of falls. Lastly and importantly, there are some advantages and disadvantages for the first two self-rated multifactorial fall risk assessment tools. Specifically, the self-rated Fall Risk Questionnaire (self-rated FRQ) is characterized as a simple and fast-screening tool, which is the fall risk screening component of the STEADI (Stopping Elderly Accidents, Deaths, and Injuries) toolkit [11]. However, Song et al. found that the Cronbach’s α of the self-rated FRQ was a bit low (0.670) when testing in the Chinese community-dwelling older adults, implying that the items in the questionnaire are not highly correlated and more relevant items are required to be added to increase its internal consistency [19]. The Chinese Home-FAST self-reported screening tool mainly focuses on assessing home environmental hazards which are classified as extrinsic factors, not paying much attention to intrinsic risks (dependent on the individualized agent) [20].

**Table 1:** The summary of fall risk assessments used for community-dwelling older adults

Scale	Author/Year	Items	Scores	Reliability	Validity	Self-assessment
**The Performance-based Tests**						
Berg Balance Scale (BBS)	Muir et al. 2008 [13]	14	0 ~ 56	NR	Sen: 61% Spe: 53% Cut-off point: 54 AUC:0.59	No
Time Up and Go test (TUGT)	Kang et al. 2017 [14]	1	Time recorded	NR	Sen: 67.5% Spe: 56.3% Cut-off point: 10.15 s AUC:0.607	No
Functional Gait Assessment (FGA)	Wrisley & Kumar 2010 [15]	10	0 ~ 30	NR	Sen: 100.0% Spe: 82.8% Cut-off point: 20 AUC:0.92	No
Dynamic Gait Index (DGI)	Wrisley & Kumar 2010 [15]	8	0 ~ 24	NR	Sen: 100.0% Spe: 75.9% Cut-off point: 20 AUC:0.91	No
**The Fall-related Psychological Evaluations**						
Activities-specific Balance Confidence (ABC) scale	Guan et al. 2012 [16]	16	0 ~ 100%	Cronbach’s α: 0.94 Inter-rater: ICC = 0.98 Test-retest: ICC = 0.96	Significant discriminatory validity (*t* = 3.45, *P* < 0.01)	Yes
Fall Efficacy Scale-International (FES-I)	Guo et al. 2015 [17]	16	16 ~ 64	Cronbach’s α: 0.921 Test-retest: ICC = 0.906	Sen: 71% Spe: 63% Cut-off point: 35 AUC:0.741	Yes
Iconographical Falls Efficacy Scale-Short Version (Icon-FES)	Chan et al. 2018 [18]	10	10 ~ 40	Cronbach’s α: 0.91 Test-retest: ICC = 0.93	Concurrent validity: correlate with FES-I (r = 0.75, *P* < 0.001)	No (Interview-based)
**The Multifactorial Fall Risk Assessment Tools**						
The self-rated Fall Risk Questionnaire (self-rated FRQ)	Song et al. 2020 [19]	12	0 ~ 14	Cronbach’s α: 0.670	Sen: 81.03% Spe: 51.72% Cut-off point: 4 AUC:0.743	Yes
The Chinese Home-FAST self-reported screening tool	Lai et al. 2020 [20]	20	0 ~ 20	Cronbach’s α: 0.94 Inter-rater: ICC = 0.89 Test-retest: ICC = 0.88	Satisfactory discriminatory validity (Wilks’ lambda = 0.78, F = 42.04, P < 0.001)	Yes
The Fall-risk Assessment Profile	Chen et al. 2020 [21]	8	0 ~ 17	NR	Sen: 75.16% Spe: 52.75% Cut-off point: 6 AUC: 0.70	No
The Short-form Physiological Profile Assessment (S-PPA)	Liu & Ng. 2019 [22]	5	NR	Inter-rater: ICC = 0.83 Intra-rater: ICC = 0.74	Sen: 39% Spe: 81% Cut-off point: 0.87 AUC: 0.62	No
The Fall Risks for Older People in the Community screening tool (FROP-Com screen)	Ng et al. 2020 [23]	3	0 ~ 9	Inter-rater: ICC = 0.79 Test-retest: ICC = 0.70	Concurrent validity: correlate with BBS (rho = 0.38, *P* < 0.01), TUG (rho = 0.35, *P* < 0.01), and ABC-C (rho = − 0.65, *P* < 0.001).	No
The Fall Risk Screening Tool	Fielding et al. 2013 [24]	23	0 ~ 33	Cronbach’s α: 0.869 Inter-rater: ICC = 0.830	NR	No
LASA Fall Risk Profile	Peeters et al. 2010 [25]	9	0 ~ 30	NR	Sen: 56.6% Spe: 71.4% Cut-off point: 8 AUC: 0.65	No

*Sen* Sensibility, *Spe* Specificity, *AUC* Area Under Curve, *NR* Not Reported

Currently, there is a lack of abundant information regarding the comprehensive fall risk self-evaluation scale for older people living in the community. Thus, it is indispensable to develop a fall risk self-assessment scale tailored to Chinese community-dwelling older adults, which is a major issue of immediate and far-reaching significance in a sense. Early chunks of research had been conducted to develop the fall risk self-assessment scale in community-dwelling older people (FRSAS) using the three-round modified Delphi method [26]. Therefore, this study aimed to evaluate the reliability and validity of the FRSAS among community-dwelling older adults.

## Methods

### Participants

This was a cross-sectional study using a convenience sampling to recruit older people between October and December 2020. A total of 230 community-dwelling older people aged 65 years and over were recruited through posters in the three local community healthcare centers of Haidian district, Beijing. Inclusion criteria of participants were: aged 65 years or older; had resided in the current community for at least 1 year; no communication barriers including written or verbal communication (based on the self-report information of participants and observation of researchers); able to ambulate independently (including with the help of assistive devices, such as cane and walking aid, etc.). Older adults were excluded who were completely immobilized (e.g., paralysis or amputation), had moderate to severe cognitive impairment or had mental disorders (judged by the self-report information of participants and observation of researchers, which indicated that participants would be required to do further assessments including MMSE if necessary). According to Kendall’s guidelines [27], the ratio of variables in the scale and samples should be no less than 5:1. The attrition rate considered, a minimum of 220 participants was determined. The study was approved by the Peking University Third Hospital Medical Science Research Ethics Committee (Ref: M2020392). Informed consent was obtained from all participants in the beginning of the study and the collected data were kept blindly.

### Procedures

This study involved two stages: 1) Development of the FRSAS and 2) Testing its reliability and validity.

#### Stage 1: development of the FRSAS

The development of the FRASA was composed of two steps. Firstly, the item pool of fall-related risk factors was compiled from a literature review or current assessments utilized commonly in practice and complemented by the recommendations of stakeholders derived from the focus group. Secondly, a three-round modified Delphi study was employed to reach a consensus within an expert panel.

In step one, a literature review was conducted by searching PubMed, Medline, Embase, Wanfang Data and China National Knowledge Infrastructure (CNKI) in the last 5 years. The main search terms were ‘older ‘or ‘aged’, ‘falls’ or ‘accidental falls’, ‘risk assessment’ or ‘risk screening’, ‘scale’ or ‘tool’ or ‘instrument’, in various combinations using a Boolean operator. Only searching results from prospective cross-sectional or cohort studies, clinical trials, reviews and meta-analysis published in English or Chinese were included for further evaluation (For the process of literature review referred to Additional file 1). The current assessments were utilized for reference, such as the Morse Fall Scale [28], Hendrich II Fall Risk Model [29] and the Home Falls and Accidents Screening Tool (HOME FAST) [30]. The items extracted from the literature review and the current assessment tools were discussed by a focus group. The inclusion criteria of invited experts in the focus group were: (1) experts specialized in rehabilitation, nursing, geriatrics or gerontology, including clinicians, nurses and physical therapy (had work experience in community healthcare center); (2) experts who had been engaged in clinical or research work for more than 5 years; (3) experts who had possessed advanced title. Finally, the two-level item pool was settled, where 5 first-level indicators and 37 second-level indicators were included.

In step two, a three-round modified Delphi process was conducted according to published procedures and guidelines between January and June 2020 [31, 32]. Twenty-nine experts recruited in the process were asked to rate the importance of first-and-second level indicators, using 5-point Likert scales with ratings from 1 (definitely not important) to 5 (extremely important) and provide suggestions or comments about their ratings.

#### Stage 2: testing its reliability and validity

Eligible participants were requested to complete the FRSAS assessment by themselves independently as much as possible. If it were challenging for participants to fill in the scale, such as the blurred vision of themselves, the caregivers or researchers would verbally repeat the items to them and tick what they chose. In other words, the choice made in each item was based on the participants’ response. Some demographic and clinical data were recorded initially, including age, gender, height, weight, marital status, educational level, living condition and residential type. Additional questions were asked regarding the fall accidents and fear of falling: “In the past 12 months, do you have any falls including a slip or trip in which you lost your balance and landed on the floor or ground or lower level?” and “generally speaking, do you have the fear of falling?”. To examine test-retest reliability, fifty participants who were selected from the total sample by using random number table were invited to complete the FRSAS assessment once more in 1 week apart. The FRSAS could be re-evaluated by an in-person or phone call.

### Instruments

The FRSAS, which is developed by our research team, constitutes 41 items in 5 dimensions: demographic characteristic, physical functions, general conditions, contexts and health-related issues and medication. All items are dichotomously rated as “Yes” if the picture described in the scale is present and “No” if the picture is not fit for the older adults. Each item can be scored as 0 or 1 depending on the question, while the scoring of item H12 rests with the number of chronic diseases identified ranging from 0 to 6. Individuals’ scores from each of the 5 dimensions are added to produce a total possible score of 46. A higher score on the FRSAS indicates a higher risk of falling.

### Statistical analysis

Data analyses were performed using SPSS version 20.0 (SPSS Inc., Chicago, IL, USA). Descriptive data were reported as frequency and percentage (%). Continuous data with normally distribution were given as mean and standard deviation (SD); otherwise, medians and 1st and 3rd quartiles were calculated. *P* < 0.05 was considered as statistical significance for the differences.

Feasibility was assessed by using the completion rate, the time taken to complete the scale and the understandability of the scale (this information was collected by the feedback of participants). Internal consistency of FRSAS was measured using Cronbach’s α. According to the literature, Cronbach’s α greater than 0.9 was considered as excellent, 0.8–0.9 as good, 0.7–0.8 as acceptable, 0.6–0.7 as questionable, 0.5–0.6 as poor and lower than 0.5 as unacceptable [33]. The test-retest reliability was examined using the intra-class correlation ICC (2,1), which was classified into four categories based on ICC value: poor (less than 0.5), moderate (between 0.5 and 0.75), good (between 0.75 and 0.9) and excellent (greater than 0.90) [34]. Absolute reliability was assessed by calculating the standard error of measurement (SEM) for the repeated measurements. The inverse relationship is presented between ICC and SEM, which indicates the higher reliability a test has, the fewer error of measurement [35].

The Bland and Altman method was performed to evaluate agreement in scores between two tests by calculating the difference score and mean score between test and retest and plotting them against each other. Acceptable agreement between the two tests was found when 95% of the mean scores fell between the limits of agreement [36]. It is suggested that the participants will score more fall risks in the initial test than the repeated if the mean difference is positive, whereas a negative mean difference indicates the opposite. Meanwhile, no difference is found in the FRSAS score between the two tests when the mean difference is zero.

Exploratory factor analysis (EFA) was applied to determine which subgroup each item could be categorized. Kaiser-Meyer-Olkin’s test and Bartlett’s test of sphericity were performed to determine the sample adequacy and the inter-variables relationship, respectively. The factor structure was explored using principal component analysis with varimax rotation. We used t-tests or Wilcoxon rank-sum test to analyze the differences in total scores and sub-dimensional scores of FRSAS between the faller and nonfaller groups. *P* < 0.05 was considered as statistical significance for the differences.

## Results

### Development of the FRSAS

Through three-round consultations, 5 first-level and 41 second-level indicators were finalized, as presented in Table 2.

**Table 2:** The first-level and second-level indicators of the FRSAS

First-level indicator	Code	Second-level indicator
Demographic (D)	D1	Age ≥ 80 years
Physical functions (P)	P1	Getting in and out of bed safely and effortlessly
	P2	Rising from the chair and sitting down safely and effortlessly
	P3	Toileting safely and effortlessly
	P4	Bathing safely and effortlessly
	P5	Reaching/Carrying the commonly used items effortlessly without inducing a fall
	P6	Stopping to pick up little items, such as pieces of paper, coin, etc.
	P7	Translating the food from kitchen to table safely and effortlessly
	P8	Comfortable and non-slip shoes
	P9	Donning and doffing pants, skirts, shoes, and socks effortlessly
	P10	Going up and down stairs smoothly (assistive device can be used, such as rails)
	P11	Stepping over obstacles smoothly
	P12	Walking with mobility aid, such as cane.
General conditions (G)	G1	Good quality of sleep
	G2	Being fatigue easily during daily activities
	G3	Going to be bathroom habitually at night
	G4	Exercising more than half an hour daily
	G5	Fear of falling
Contexts (C)	C1	Flat ground at home
	C2	Debris clustered on the ground
	C3	Moveable mat or carpet unsteadily
	C4	Water residual on the ground frequently
	C5	Switching on and off the light conveniently in the bed
	C6	Well-lit corridor, house, and community
	C7	Immobilized non-slip mat in the bathroom
	C8	Handrails installed in the bathroom
	C9	Residing in a bungalow or walk-up building
	C10	Living alone
	C11	Understanding fall prevention heretofore
Health-related issues and medication (H)	H1	Dizziness and vertigo
	H2	Arthrodynia of lower extremity
	H3	ROM (Range of motion) limitation or deformity of lower extremity
	H4	Vision problems
	H5	Toileting frequently for bowel and bladder dysfunction
	H6	Numbness and pain in lower extremities for cervical and/or lumbar disease
	H7	History of brain injury and cerebrovascular accident (CVA)
	H8	Taking antiepileptic drug (AED)
	H9	Taking antipsychotic
	H10	Fall histories
	H11	Excessive drinking daily (the average of alcohol consumption more than 25 g per day for male, which equals to 750 ml beer, 250 ml wine, 75 g liqueur with 38°and 50 g highly liqueur, while the threshold is 15 g for female)
	H12	Chronic disease (Tick where relevant): Alzheimer’s Disease Parkinsonian Syndrome Coronary Heart Disease Chronic Obstructive Pulmonary Disease (COPD) Osteoporosis Diabetes Mellitus

### The demographics of participants

Of the 230 participants recruited in the study, 222 participants completed the assessment as required through two-round quality check. Among the 222 qualified participants, 82 were male accounting for 36.94% and 140 females representing 63.06% of the total. The mean age of participants was 73.84 ± 7.46 years, ranging from 65 to 90 years. Around 30% of older adults reported a history of falls in the past 12 months. Meanwhile the number of participants who had a fear of falling doubled to those who had not. The demographic information of participants was enlisted in Table 3.

**Table 3:** Demographics of participants (*n* = 222)

Project	Category	Frequency (N)	Percentage (%)
Sex	Male	82	36.94
	Female	140	63.06
Age (years)	65 ~ 74	130	58.56
	75 ~ 84	62	27.93
	≥85	30	13.51
BMI (kg/m^2^)	<18.5	8	3.60
	18.5 ~ 23.9	97	43.69
	24.0 ~ 27.9	83	37.39
	≥28	34	15.32
Marital status	Unmarried	2	0.90
	Married	180	81.08
	Divorced	2	0.90
	Widowed	38	17.12
Educational level	Primary school or below	23	10.36
	Secondary school	58	26.13
	High school	55	24.77
	Junior college	41	18.47
	Undergraduate	41	18.47
	Master or above	4	1.80
Living condition	Alone	28	12.61
	With family members	194	87.39
Residential type	Bungalow	8	3.60
	Walk-up building	119	53.60
	Building with elevator	95	42.79
Fear of falling	Yes	159	71.62
	No	63	28.38
History of falls in the past 12 months	Yes	73	32.88
	No	149	67.12

### Feasibility

The completion rate of the FRSAS was 96.52% and the FRSAS could be completed in 10 min. The majority of items on the FRSAS could be easily understood without ambiguity. Based on the feedback of the respondents, some ambiguous items should be modified or elaborated to reach a consensus. For example, item P11 “stepping over obstacles smoothly” can be parenthetically noted the characteristics of obstacles (e.g., the height and width) and item G1 “good quality of sleep” can be appended with some explanatory descriptions to explicate the good quality of sleep. Overall, the FRSAS had relatively high feasibility.

### Internal consistency

The internal consistency of the entire FRSAS was acceptable with Cronbach’s α = 0.757. Cronbach’s α if item deleted ranged between 0.746 and 0.768. When removing eight items in which Cronbach’s α if item deleted was lower than 0.757, the total Cronbach’s α rose to 0.800.

### Test-retest reliability

Fifty older adults participated in the retest of FRSAS by either in-person or phone call. The standard error of measurement (SEM) of the FRSAS was 0.186. The distribution of difference scores between test and retest were conformed to a normal distribution (determined by the Shapiro-Wilks test). The overall ICC (2,1) value for the test-retest reliability was 0.967 (Table 4).

**Table 4:** Results of analyses of test–retest reliability

	Test 1			Test 2					
*N* = 222	Mean	SD	Range	Mean	SD	Range	ICC (2,1)	95%CI	SEM
FRSAS	13.54	5.02	6 ~ 26	13.12	5.12	5 ~ 25	0.967	0.943 ~ 0.981	0.186

*FRSAS* Fall Risk Self-Assessment Scale, *SD* Standard Deviation, *ICC (2,1)* Intra-Class Correlation, *SEM* Standard Error of Measurement

**Fig. 1:**
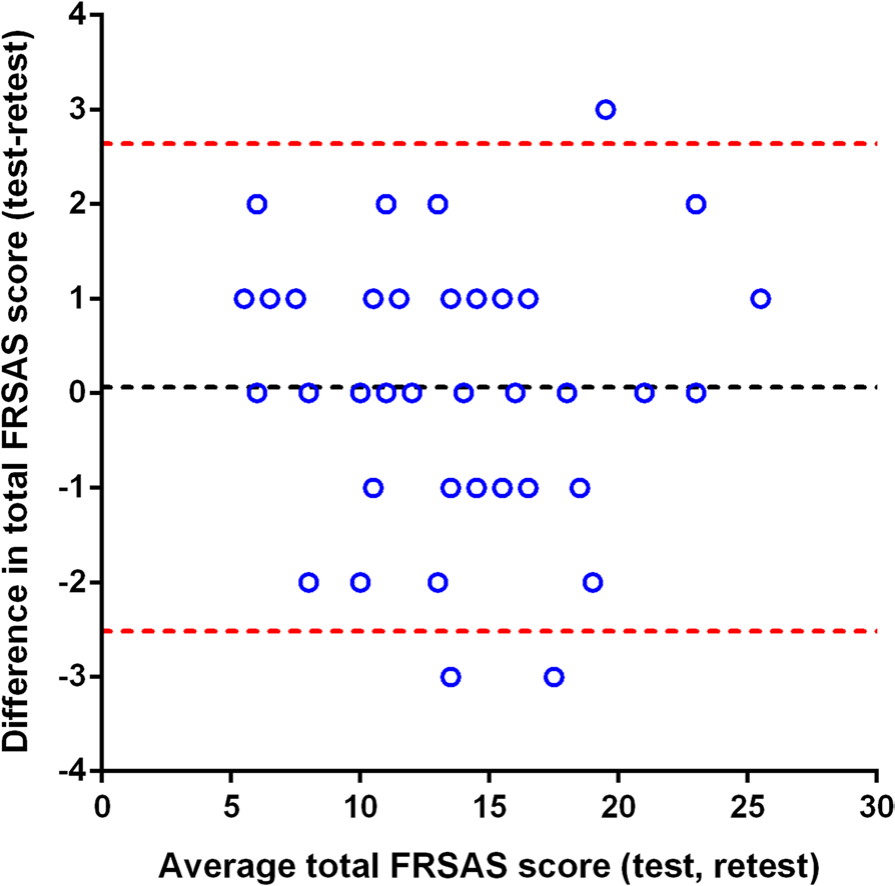
Bland and Altman plot on the agreement between test and retest

A minor positive mean difference (Mean = 0.06) yielded on FRSAS scoring between the initial and repeated assessments and 95% of the difference score fell between the limits of agreement (95% LoA: −2.519 ~ 2.639), indicating that the consistency of the scoring was obtained over time as depicted in Fig. 1.

### Construct validity

The KMO’s value was 0.728 (value higher than 0.7 was considered as acceptable) and Bartlett’s test of sphericity was significant (P < 0.001), both of them suggested that factor analysis was appropriate to analyze the data [37]. According to Kaiser criterion [38], 14 factors with eigenvalues greater than 1 were extracted, explaining 61.744% of the total variance. Items were considered to fall in each factor if its factor loading was 0.40 or above [39]. However, five items consisting of G3, C1, C7, H1 and H5 did not fit for any factor with its factor loading ranging from 0.347 to 0.397.

### Discriminant validity

**Fig. 2:**
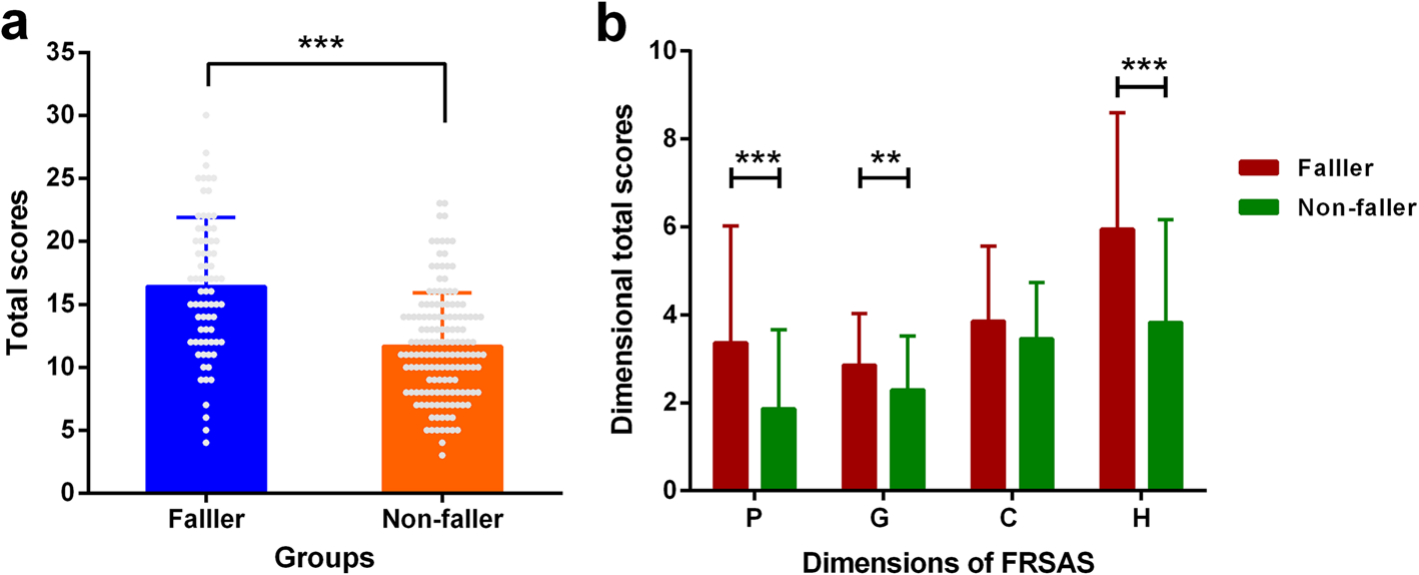
The difference of scores between faller and non-faller groups. **a** The difference of total scores between faller and non-faller groups. **b** The difference of dimensional total scores between faller and non-faller groups. ** *p* < 0.01, *** *p* < 0.001

The FRSAS total score was significantly higher for older adults who have fallen than for older adults who have not fallen in their past 12 months (Fig. 2a). When we compared dimensional total scores (except for D dimension, because it was not distributed normally) between faller and non-faller groups, the average dimensional score was significantly higher in those with a history of fall apart from C-dimension (Fig. 2b). There was no difference in the C-dimensional scores between the faller and non-faller groups (t = 1.809, *P* = 0.073). Our results showed a significant difference in D-dimensional scores between the two groups by Wilcoxon rank-sum test (Z = 2.248, *P* < 0.05).

## Discussion

The development of the FRSAS including five dimensions (i.e., the demographic, physical functions, general conditions, contexts, health-related issues and medications) is tailored to the Chinese geriatric-characteristic culture, which is reflected in the specific operational definition of each item. The FRSAS, characterized by its simplicity, understandability, fast-screening and administrator-free, can evaluate the intrinsic and extrinsic risk factors of falls comprehensively. Furthermore, our findings indicated that the FRSAS had relatively good levels of reliability and validity, signifying that this tool could be utilized to assess fall risk in the community-dwelling Chinese older population.

The results showed that the internal consistency of the FRSAS was acceptable (Cronbach’s α = 0.757). If eight items (Cronbach’s α became higher than the previous one when deleting the item) were removed, Cronbach’s α increased to 0.800. Neither a low Cronbach’s α nor a very high Cronbach’s α indicates an acceptable correlation between the items in the scale [40]. The former makes the items unjustified. The latter manifests the redundancy of one or more items. Compared with the self-rated Fall Risk Questionnaire (Cronbach’α =0.670) and the Chinese Home-FAST self-reported screening tool (Cronbach’α =0.940) with regard to the internal consistency [19, 20], the Cronbach’α of the scale obtained in this study was good, indicating that each item of the scale has a good homology.

The Intraclass Correlation Coefficient ICC (2,1) of FRSAS was 0.967 (95%CI: 0.943 ~ 0.981), indicating its excellent reproducibility. It is noteworthy that the ICC (2, 1) is very sensitive and directly impacted by the intra- and inter-subject variability [41]. The FRSAS scores also showed good agreement based on the SEM value and Bland-Altman plot for both tests in the one-week interval. Ng et al. reported that the ICC of the Falls Risk for Older People in the community screening tool was only 0.70 [23]. Likewise, Lai et al. found that ICC of the Chinese Home-FAST self-reported screening tool was 0.88 [20]. Both suggested that the FRSAS had good reproducibility.

According to the exploratory factor analysis, the whole scale was divided into 14 factors. The criterion used in this study to determine how many factors to retain is the Kaiser criterion, which proposed that an eigenvalue greater than 1.0 is a good lower bound for expecting a factor to be meaningful [38]. Controversially, the consensus was reached in the literature that the Kaiser criterion probably gets less accurate as more items are analyzed [42]. Based on this, researchers suggested that the Kaiser criterion can be employed in conjunction with examination of the scree plot for deciding the number of factors to extract in an exploratory factor analysis [43]. However, Ruscio & Roche pointed out that both the Kaiser criterion and scree plots have been shown to overestimate the number of dimensions in data. In the future study, it is recommended that taking advantage of parallel analysis (PA) supplemented by comparison data (CD) can help researchers model their data more precisely and ultimately to develop more reliable and valid assessment instruments [44].

In term of the discriminant validity, the FRSAS scores in the faller group were significantly higher than the non-faller group, verifying our predefined hypotheses that FRSAS would show greater scores among older individuals who have fallen in the previous 1 year than among those who have not. Likewise, the inherent dimensions also yielded the same result except for C-dimension (i.e., contexts). Non-significantly statistical differences for the C-dimension might be explained from following aspects. First of all, the samples included in this study were the community-dwelling older population in Beijing, whose living environment had common problems such as old floors and narrow space, resulting in the consequence that the distinction between falling and the non-falling older population was not high. If older adults in other areas (e.g., western, eastern, southern, or central regions of China) were included, the scores of contextual dimensions might show statistical differences due to the change in a living environment. Secondly, since older adults were gathered in the specific place for fall risk assessment, the identification of home hazards in the scale mainly depended on retrospective information rather than observation in real-life environments and contexts, which caused the insignificant difference of contextual dimension in the older population with or without falling. Finally, home hazards could not be efficaciously reflected using the 11 items in the scale. Some tools that existed for home hazards evaluation had proven to be valid. The Chinese HOME FAST self-reported screening tool provided satisfactory discriminatory ability concerning unplanned fall incidents [20]. However, the items in this self-reported screening tool are not as simple as the one we developed, which causes that more time is required for older adults to consolidate their identification of home hazards. Overall, we can use it as reference to enhance the differentiation of the contextual dimension of this scale in the group with or without falls.

The strength of this study is that it is the first study to develop a FRSAS accommodated to Chinese geriatric-characteristic culture and evaluate its reliability and validity among community-dwelling older people. FRSAS once developed successfully, can assist the clinicians or general healthcare providers for fall prevention among older people living in a community setting. However, it must be taken into account that some limitations were not avoided in this study. First of all, some inclusion and exclusion criteria were dependent on the self-reported information of participants, such as no communication barrier, moderate to severe cognitive impairment or mental disorders. Secondly, all the participants were recruited from three communities in Beijing by the convenience sampling strategy, causing that the sample could not be completely representative of the whole population and the results in the pilot study could not be generalized to the whole population. Thirdly, since this was a cross-sectional study, falling information was taken retrospectively, which caused the information bias to some extent. Furthermore, the assessment of falls and fear of falling were self-reported by participants. Lastly, the criterion validity and the responsiveness of FRSAS was not detected.

## Conclusions

To sum up, the FRSAS including 41 items demonstrated relatively good feasibility as well as satisfactory results in the internal consistency, test-retest reliability, and discriminant validity. However, some concerns had arisen regarding the construct validity in the pilot study. Adaptation of the FRSAS is the next stage of our future work, followed by testing in different types of population using larger sample sizes as well as providing a more convenient and fast-screening tool for Chinese community-dwelling older people.

## Supplementary Information

**Additional file 1.** The process of literature review.

## Data Availability

The datasets used and/or analyzed during the study are available from the corresponding author or first author on reasonable request.

## Abbreviations

FRSAS: The Fall Risk Self-Assessment Scale
D: Demographic Characteristics
P: Physical Functions
G: General Conditions
C: Contexts
H: Health-related Issues and Medication
Sen: Sensibility
Spe: Specificity
AUC: Area Under Curve
BBS: Berg Balance Scale
TUG: Time “Up & Go” test
ABC-C: Chinese version of the Activities-specific Balance Confidence Scale
HOME FAST: The Home Falls and Accidents Screening Tool
SD: Standard Deviation
ICC: Intra-Class Correlation
SEM: Standard Error of Measurement
95% LoA: 95% Limits of Agreement
EFA: Exploratory Factor Analysis
KMO: Kaiser-Meyer-Olkin
