# Supplementary material for: Reliability and validity of the fall risk self-assessment scale for community-dwelling older people in China: a pilot study
Source: BMC Geriatr. 2022 Apr 1;22:272. doi: 10.1186/s12877-022-02962-3 (PMC8976342; doi:10.1186/s12877-022-02962-3)
Supplement: Supplementary file 1 — Additional file 1. The process of literature review. [file 12877_2022_2962_MOESM1_ESM.pdf]

Additional file 1: The process of literature review

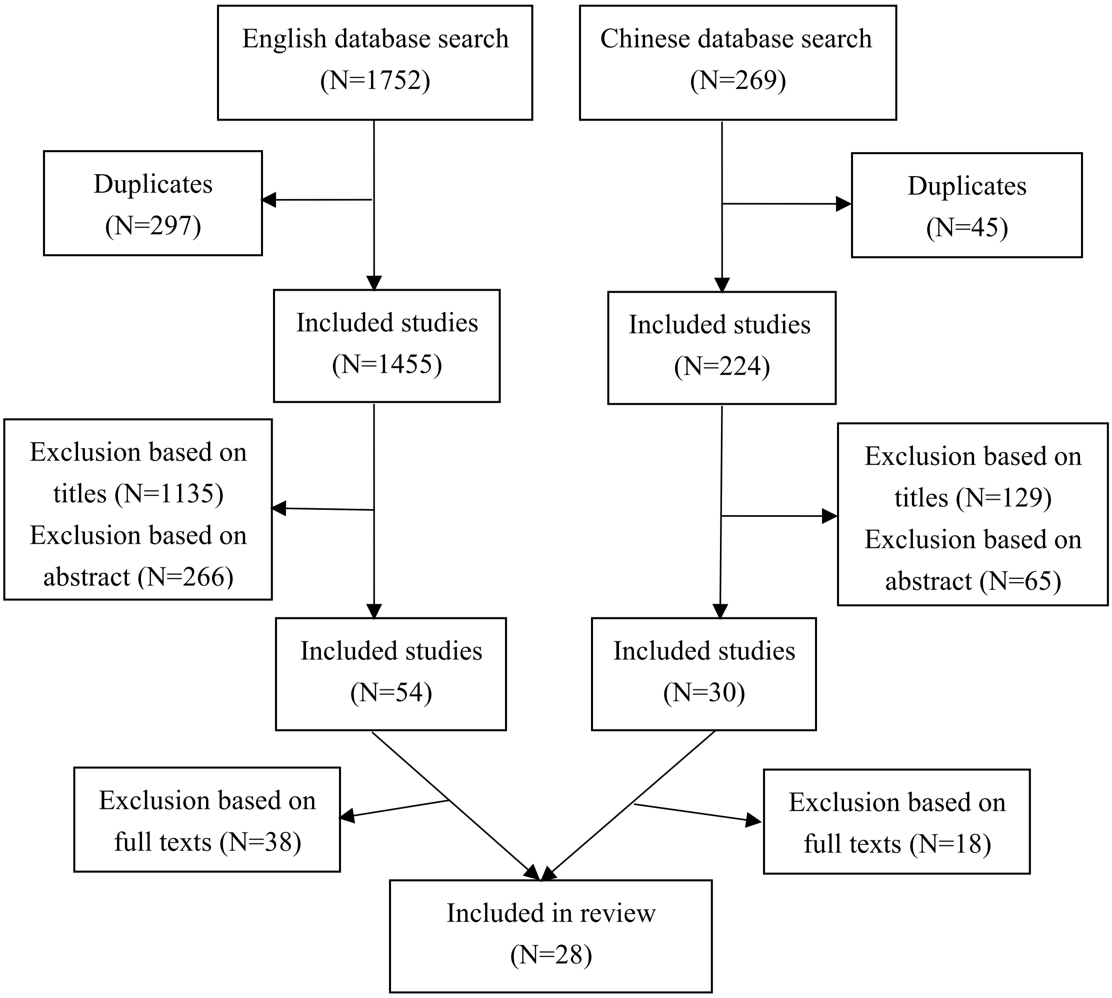

Figure S1. Flowchart of the literature review process
